# Supplementary material for: Phytosterols Are Involved in Sclareol-Induced Chlorophyll Reductions in Arabidopsis
Source: Plants (Basel). 2023 Mar 11;12(6):1282. doi: 10.3390/plants12061282 (PMC10055023; doi:10.3390/plants12061282)
Supplement: Supplementary file 1 [file plants-12-01282-s001.zip › plants-2110192-supplementary.pdf]

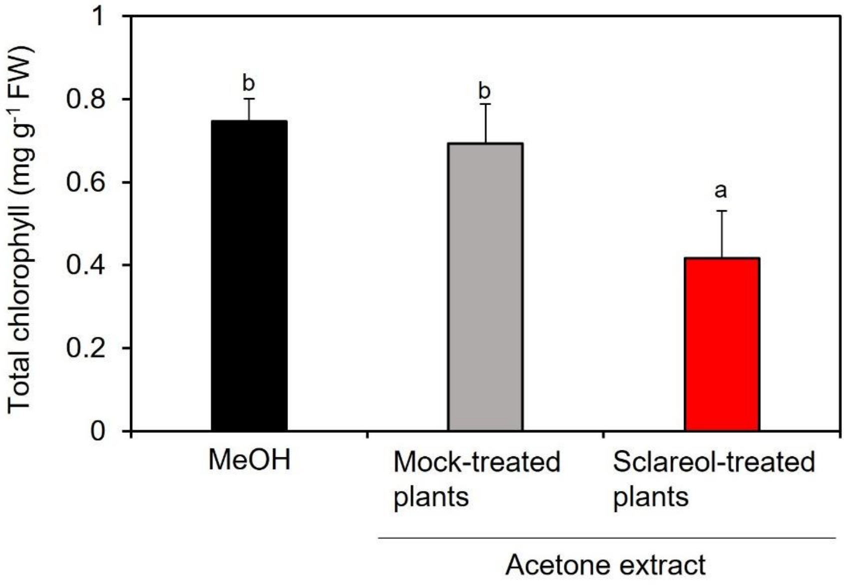

Supplementary Figure S1. The effect of acetone extracts prepared from *Arabidopsis* plants on the accumulation of chlorophyll. Four-week-old *Arabidopsis* plants were treated by soaking their roots in a solution containing 100 μM sclareol or 0.1% methanol (Mock) for 48 h. The leaves of the treated plants were collected and subjected to the acetone extraction system. Each acetone extract was applied to Col-0 plants, and the contents of total chlorophyll, represented as the sum of chlorophyll *a* and *b*, were measured. As a control, 0.1% methanol (MeOH) was used. Values are the means ± standard deviation of three biological replicates. Different letters indicate significant differences among treatments (Tukey's test, $P < 0.05$).

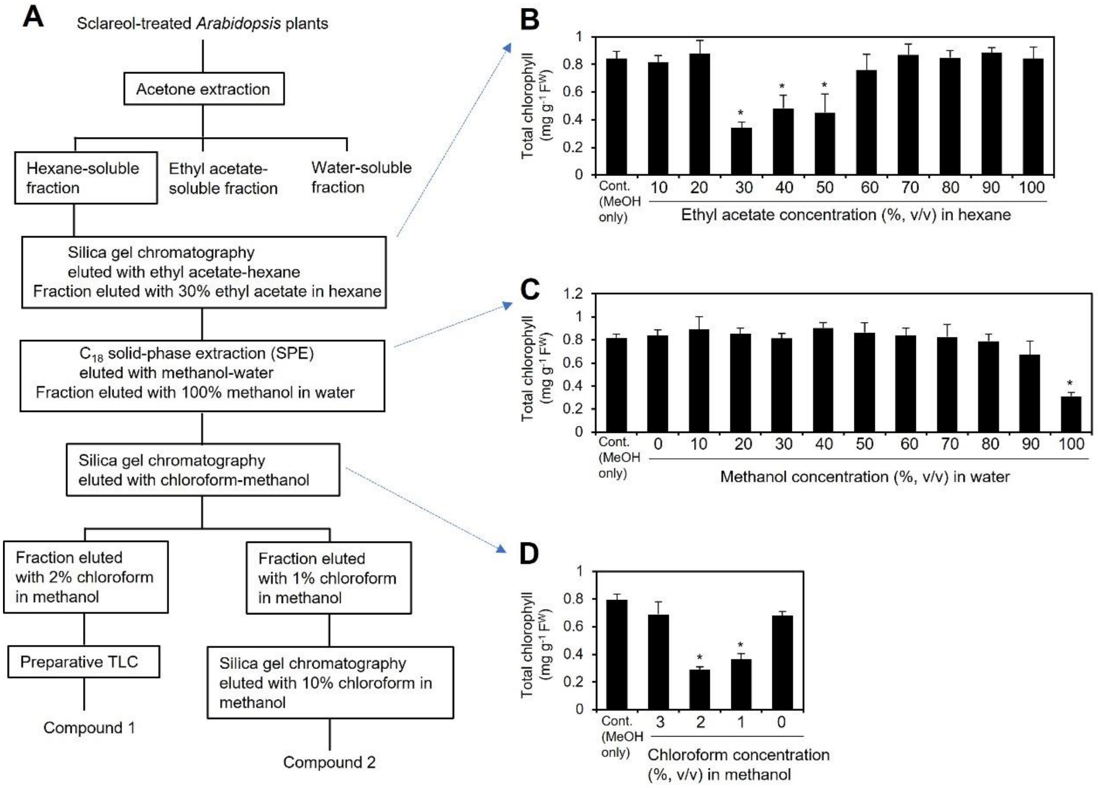

Supplementary Figure S2. Flow diagram for purification and isolation of chlorophyll content-reducing substances from *Arabidopsis* plants. (**A**) Extraction and purification procedure. (**B**) Chlorophyll content-reducing activity in fractions obtained by first silica gel column chromatography. (**C**) Chlorophyll content-reducing activity in fractions obtained by C_{18} SPE chromatography. (**D**) Chlorophyll content-reducing activity in fractions obtained by second silica gel column chromatography. For **B** to **D**, 0.1% methanol (MeOH) was used as a control. Values for **B** to **D** are the means ± standard deviation of three biological replicates. Asterisks denote significant differences from the 0.1% methanol sample (Dunnett's test, P <0.05).

Table S1. List of primers used for the quantitative real-time PCR analysis.

Gene name	AGI code	Forward	Reverse
AtPDR12	At1g15520	CTTTCGCTCAGGTTTTCATCG	CTTCACCGCCGTCCACTC
AtHMGR	At1g76490	CCTGCTGCTGTGAACTGGATT	TTGTTCAC-GATCTCTCCTCTGATT
AtSMO1-2	At4g22756	CGTTGGAGGACAAAGCCAGA	CCAGCTTGTTGCTCTTCTTGG
AtDWF1	At3g19820	GCAGCCAATCAAAGGCCAAA	GCCAGGTGCGTAGTAGACTC
Atactin2	At3G18780	GGTAACATTGTGCTCAG-TGGTGG	GGTGCAACGAC-CTTAATCTTCAT
